# Serum metabolite levels identify incipient metastatic progression of rectal cancer

**DOI:** 10.1038/s43856-025-00868-w

**Published:** 2025-04-27

**Authors:** Kine M. Bakke, Paula A. Bousquet, Sebastian Meltzer, Tonje Bjørnetrø, Frode Rise, Alistair L. Wilkins, Kathrine Røe Redalen, Anne Hansen Ree

**Affiliations:** 1 Department of Oncology, Akershus University Hospital, Lørenskog, Norway; 2 Department of Physics and Computational Radiology, Oslo University Hospital, Oslo, Norway; 3 Department of Chemistry, University of Oslo, Oslo, Norway; 4 School of Science and Engineering, University of Waikato, Hamilton, New Zealand; 5 Department of Physics, Norwegian University of Science and Technology, Trondheim, Norway; 6 Institute of Clinical Medicine, University of Oslo, Oslo, Norway

**Keywords:** Cancer metabolism, Diagnostic markers, Metastasis

## Abstract

**Background:**

The cellular metabolism undergoes reprogramming during the metastatic process. We hypothesised that serum metabolites at the time of primary tumour diagnosis might identify rectal cancer patients prone to metastatic progression.

**Methods:**

One hundred twenty-three rectal cancer patients from a prospective observational biomarker study were followed up to 5 years after study entry. We have assessed metabolites in serum sampled at the time of diagnosis by ^1^H-nuclear magnetic resonance spectroscopy, using the internal reference trimethylsilylpropanoic acid for quantification.

**Results:**

Here we show that patients who develop overt metastatic disease more than 6 months after the primary tumour diagnosis have elevated serum levels (Kruskal-Wallis test) of alanine (*P* = 0.005), lactate (*P* = 0.023), pyruvate (*P* = 0.041) and citrate (*P* = 0.007) compared to those without metastases at the 5-year follow-up or with metastases already 6 months or sooner after the cancer diagnosis. Patients with serum citrate above 0.24 mmol/L have poorer progression-free survival compared to those with levels below (*P* < 0.001; log-rank test).

**Conclusions:**

We observe a distinct serum metabolite profile, in particular involving citrate to the best of our knowledge shown for the first time clinically, in rectal cancer patients at heightened risk of metastasis already when the primary tumour is diagnosed, offering insights into the metabolism of metastatic progression.

## Plain Language Summary

Cancer cells need energy and building blocks, such as amino acids and lipids, when growing. This is particularly important when the cancer cells are spreading to other organs in the body, named metastasis. Metastatic spread is a common cause of death from bowel cancer. We analysed metabolites (energy and building block proteins) in the blood from 123 patients with bowel cancer and found that patients who later developed metastasis had other levels of blood metabolites than patients who never were diagnosed with metastasis. In particular, we found higher levels of four specific metabolites: lactate, pyruvate, alanine and citrate. These findings may enable more precise diagnosis of bowel cancer at risk of spread to other organs and may help us better understand why metastasis occurs.

## Abstract (web summary)

Bakke, Bousquet et al. identify serum metabolite biomarkers at the time of primary tumour diagnosis in rectal cancer participants prone to metastatic progression. These individuals show higher serum metabolites of alanine, lactate, pyruvate, and citrate compared to those without metastatic progression.

## Introduction

In rectal cancer, systematic improvements of treatment strategies have resulted in curative outcome for a significant percentage of patients with the disease localised in the pelvic cavity^1^. Still, patients may present with a localised primary tumour that biologically is at high risk of metastatic dissemination to distant organs and hence, shortening the survival.

Reprogramming of the cellular metabolism is recognised as a hallmark of cancer^2^. For cancer progression to metastasis, this consists of dynamic adaptations at every step of the metastatic cascade^3^. One of the crucial functions of reprogramming is to meet the cancer cells’ early need for the synthesis of macromolecules (proteins and lipids) in tumour proliferation and metastasis^4^.

Identifying rectal cancer patients at heightened risk of metastasis already when the primary tumour is first diagnosed may help selecting those who require intensified treatment for improved long-term outcome. Serum metabolomics has emerged as a non-invasive tool for comprehensive metabolic profiling, both to improve the biological understanding of cancer progression and as a next step in precision oncology. We hypothesise that metabolites measurable in the circulation at the time of diagnosis, using ^1^H-Nuclear Magnetic Resonance (NMR) spectroscopy, may identify rectal cancer patients prone to metastatic progression. In this paper, we show that patients progressing to metastatic disease have a distinct serum metabolite profile that in particular comprises elevated pyruvate, alanine, lactate and citrate.

## Methods

### Patients and procedures

The patients participated in a prospective observational biomarker study, where 192 individuals with suspected rectal cancer (unselected all-comers, making up a population-based cohort) were enroled between October 2013 and December 2017 at Akershus University Hospital (Lørenskog, Norway), which as the largest hospital in Norway covers more than 10% of the nation’s population. Eligible patients were 18 years or older and had no prior rectal cancer treatment. Of all enroled patients, 169 had histologically confirmed adenocarcinoma (with unknown somatic mutations since the molecular tumour profile does not have therapeutic consequence unless the patient has already developed metastatic disease). Of these, 43 patients lacked serum samples, and for 3 patients we obtained a technically defective serum ^1^H-NMR spectrum, resulting in 123 subjects for the present analysis (with adequate serum specimens collected at the time of diagnosis). The patients were followed for 5 years after study entry. In line with established practice, synchronous metastases were defined as radiologically recognised metastatic disease occurring within 6 months of the time of diagnosis, while metastatic disease developing later than 6 months was defined as metachronous. The study was approved by the Regional Committee for Medical and Health Research Ethics of South-East Norway (reference number 2013/152) and the institutional review board, and conducted in accordance with the Helsinki Declaration. All patients provided written informed consent. The study is registered with ClinicalTrials.gov (NCT01816607).

### Serum samples

The preparation of serum samples was conducted in accordance with previously reported procedures^5^, with minor adjustments. Immediately upon collection, the samples were stored at –80 °C and subjected to cryopreservation until ^1^H-NMR analysis. All the ^1^H-NMR measurements were performed over a period of three months following the full patient accrual. The frozen samples were thawed at room temperature. 200 µL of serum were combined with methanol (MeOH) in a 1:2 (v/v) ratio. The mixture was vortexed for 30 s and subsequently incubated at –20 °C for 20 min before it was centrifuged at 13,000 *g* for 15 min to precipitate proteins. The supernatant was carefully transferred to a fresh vial and dried using vacuum concentration for 4 h. The dried samples were reconstituted in 500 µL of filtered phosphate buffer containing 25 µM of the internal reference compound, trimethylsilylpropanoic acid (TSP; Sigma‐Aldrich, St. Louis, MO, USA), made in one batch and used for all sample measurements. Due to limited sample volumes, we conducted the analysis using TSP as an internal standard for chemical shift calibration and signal normalisation in a single run for each sample. Prior to ^1^H-NMR analysis, 55 µL of deuterium oxide (D_2_O; Sigma, Cambridge Isotope Laboratories, Inc., Tewksbury, MA, USA) was added to the solution. The prepared samples were subsequently loaded into 5‐mm ^1^H-NMR tubes for immediate acquisition. The personnel who performed the laboratory analyses were blinded to the metastatic status during the actual measurements, because this status was not apparent before the end of patient follow-up.

### ^1^H-NMR spectroscopy

^1^H-NMR spectra were obtained using a Bruker AVIIIHD800 spectrometer fitted with a cryoprobe and the Topspin’s (version 3.6; Bruker, Germany) cpmgpr1d pulse programme with spectral width (SW) = 14 ppm, time domain (TD) = 65536 points, data points size of real spectrum (SI) = 131072 points, relaxation delay (D1) = 4 s for HOD line presaturation and L4 = 128 and saturation time (D20) = 300 μs for T2-modulated suppression of macromolecule signals^6^. Peak identifications were established from a combination of 1D and 2D spectra and comparisons with database spectra from the Human Metabolome Database (HMDB). The 2D spectra were T2-modulated 2D correlation spectroscopy determined with D1 = 2 s for HOD line presaturation and L4 = 128 and D20 = 300 μs for T2-modulated suppression of macromolecule correlations, and total correlation spectroscopy determined with an 80 ms mixing time and Excitation Sculpted suppression of HOD signals. All samples were analysed at room temperature.

### Data pre-processing

Phase correction and baseline correction, as well as shifting the baseline ppm using the TSP peak at *δ* = 0.0 ppm as reference, were done in Topspin before the spectra were exported to Matlab (Mathworks, MA, USA) for processing. The processing consisted of the removal of the water peak. To ensure the best possible alignment of all the identified peaks for all patients, we corrected alignment of individual peaks influenced by pH differences using Icoshift^7^. Quantification was done by integrating over subsequent peaks and referencing to the TSP peak, accounting for the number of protons corresponding to each peak. Information about number of protons for the different peaks was acquired from the HMDB database. Of note, this quantification assumes that the T2-relaxation of the TSP internal standard and the metabolites are comparable.

### Statistics

All statistical procedures were done in SPSS (version 29). Most metabolites were not normally distributed, as determined by the Shapiro-Wilk test, so median values and non-parametric statistical tests were used. For group comparisons, the Mann-Whitney U test was used when comparing two groups, and the Kruskal-Wallis test was used to compare more than two groups. To visualise group differences by heatmaps, the individual metabolite values were z-normalised. Differences in progression-free survival (PFS) between patient groups were assessed by the log-rank test and visualised by the Kaplan-Meier method. Potential correlations between serum factors were analysed by Spearman’s rank correlation.

### Reporting summary

Further information on research design is available in the Nature Portfolio Reporting Summary linked to this article.

## Results

### Patients and serum ^1^H-NMR profiles

**Fig. 1:**
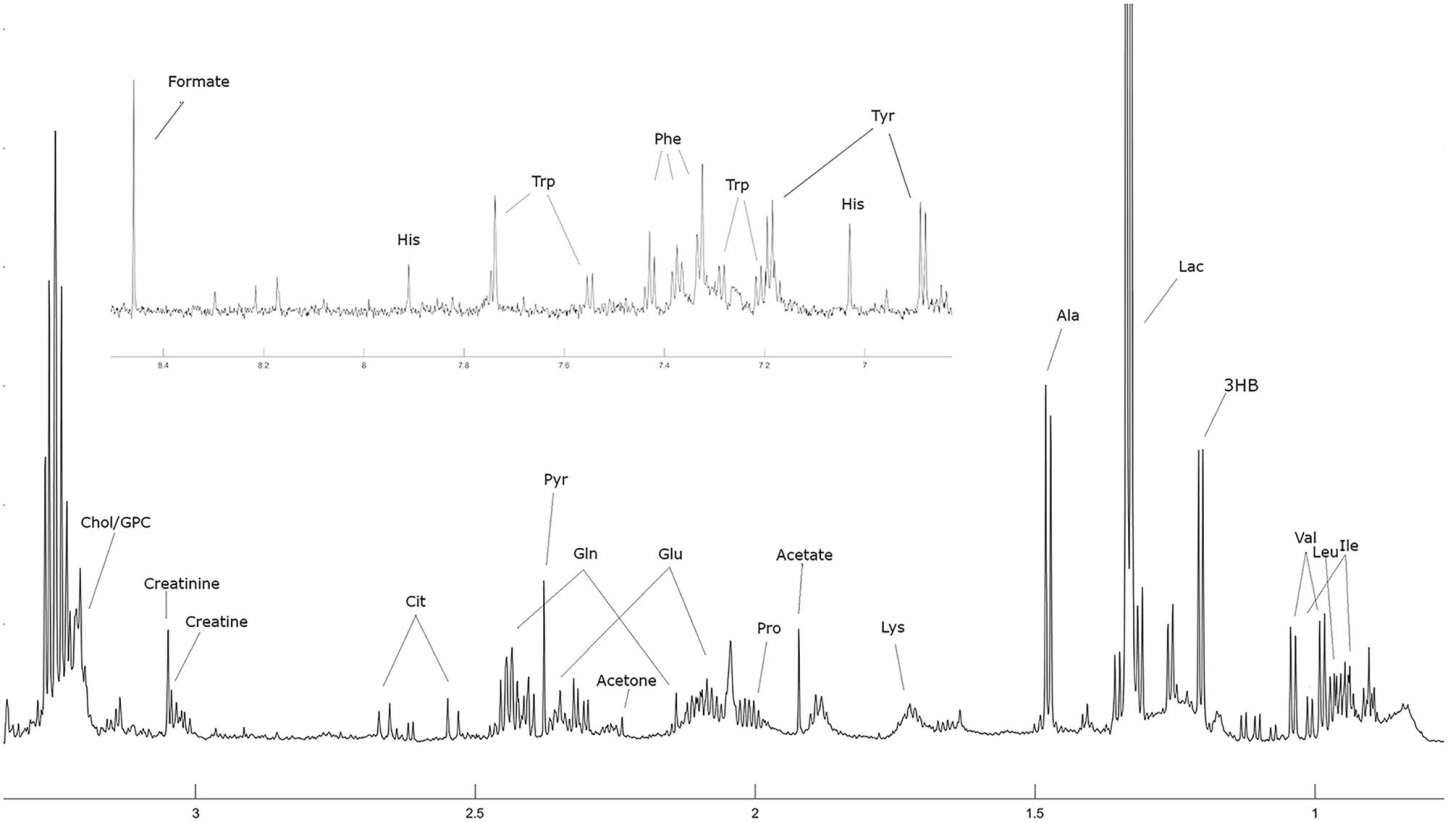
A representative ^1^H-nuclear magnetic resonance spectrum of a serum sample. The spectrum was obtained at 800 MHz. Note that the area containing the suppressed water spectra and the glucose region has been omitted. The aryl region from approximately 6.8 ppm to 8.5 ppm is superimposed above. 3HB 3-hydroxybutyrate, Ala alanine, Chol choline, Cit citrate, GPC glycerophosphocholine, Gln glutamine, Glu glutamate, His histidine, Ile isoleucine, Lac lactate, Leu leucine, Lys lysine, Phe phenylalanine, Pro proline, Pyr pyruvate, Trp tryptophan, Tyr tyrosine, Val valine.

Table 1 summarises patient characteristics, the disease at presentation (stage), which is the strongest known factor of metastatic risk, and the outcome with regard to metastatic progression for the 123 subjects from the prospective observational biomarker study. The patients were followed for 5 years after study entry for metastatic progression. Figure 1 shows a sample spectrum with annotated peaks; the patient belonged to the metachronous metastases category. A comparison of all the ^1^H-NMR profiles is shown in Supplementary Fig. 1. Table 2 lists the quantified metabolites in the 123 serum samples.

**Table 1:** Patient and disease characteristics (*n* = 123)

		Total cohort (*n* = 123)	No metastases (*n* = 78)	Metachronous metastases (*n* = 16)	Synchronous metastases (*n* = 29)
		*n* (%)	*n* (%)	*n* (%)	*n* (%)
Mean age (SD), years		65 (11)	62 (11)	67 (8)	65 (10)
Sex	Female	44 (35.8)	27 (34.6)	3 (18.8)	14 (48.3)
	Male	79 (64.2)	51 (65.4)	13 (81.3)	15 (51.7)
Stage at diagnosis (AJCC TNM)	I	29 (23.5)	25 (32.1)	4 (25.0)	0 (0)
	II	29 (23.5)	23 (29.5)	5 (31.3)	1 (3.4)
	III	38 (30.9)	30 (38.5)	7 (43.8)	1 (3.4)
	IV	27 (22.0)	0 (0)	0 (0)	27 (93.1)

*AJCC* American Joint Committee on Cancer, *SD* standard deviation, TNM tumour-node-metastasis.

**Table 2:** The quantified serum metabolites

Metabolite	Median (mmol/L)	Interquartile range (mmol/L)
Leucine	0.67	0.20
Isoleucine	0.11	0.18
Valine	0.18	0.08
3-Hydroxybutyric acid	0.40	0.36
Lactate	5.73	2.36
Alanine	0.96	0.33
Lysine	0.74	0.21
Acetate	0.32	0.11
Proline	1.52	0.52
Acetone	0.13	0.06
Glutamate	0.80	0.30
Pyruvate	0.13	0.06
Glutamine	1.18	0.33
Citrate	0.21	0.06
Creatine	0.08	0.04
Creatinine	0.02	0.06
Choline	Not quantifiable	
α-Glucose	2.32	0.81
Tyrosine	0.64	0.26
Histidine	0.06	0.03
Phenylalanine	0.19	0.06
Tryptophan	0.06	0.05
Formate	0.25	0.06

### Serum metabolic profiles and the metastatic phenotype

**Fig. 2:**
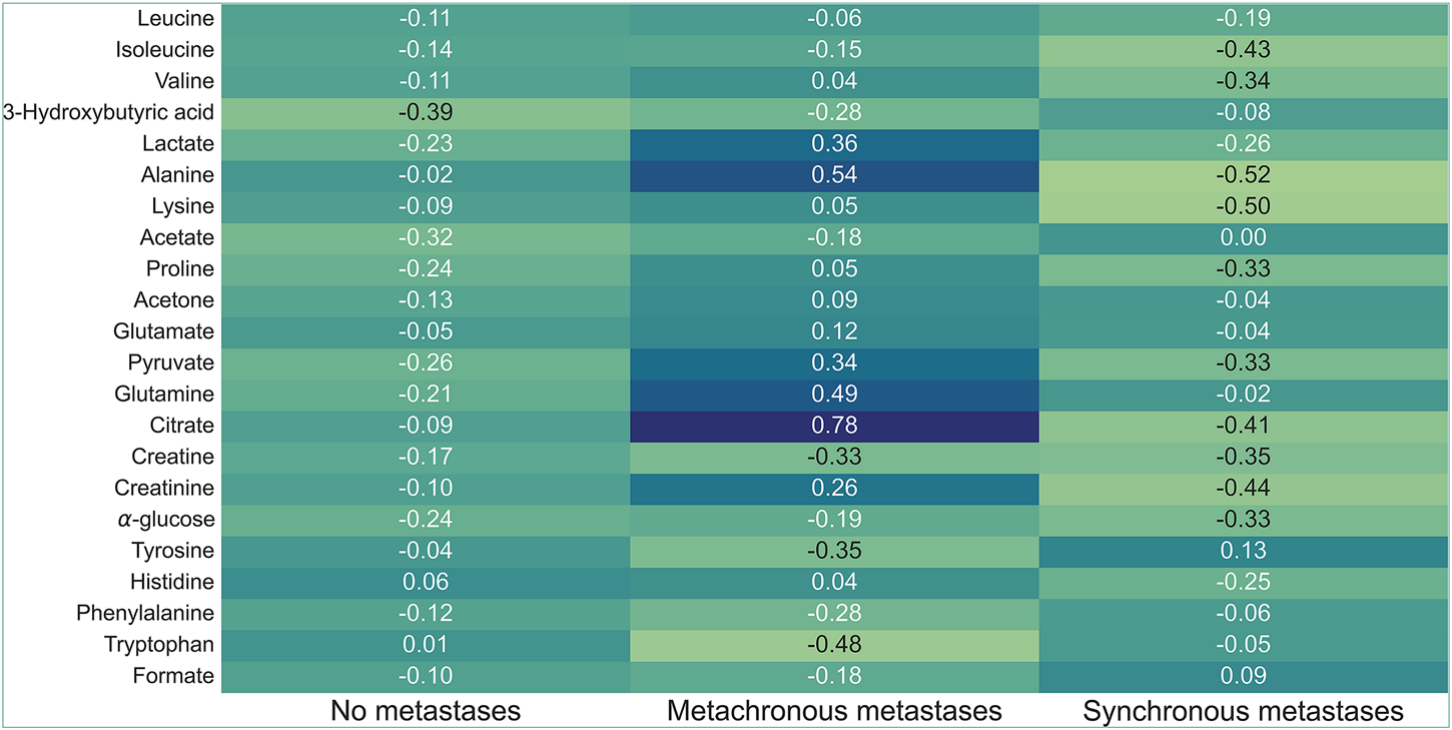
Serum metabolites according to metastatic outcome. The relative median metabolite values are shown for patients with no (*n* = 78), metachronous (*n* = 16) or synchronous (*n* = 29) metastases. The individual metabolites have been z-normalised for the colour variation to illustrate the differences between the patient groups.

To explore the metabolic profile of rectal cancer patients who might be at high risk of metastatic progression at the time of primary tumour diagnosis, and consequently could be given intensified therapy to prevent the evolving adverse process, we applied the patient categories of the definitive metastatic outcome—no metastases at the 5-year follow-up (*n* = 78), metachronous metastases (*n* = 16) and synchronous metastases (*n* = 29). The metachronous metastases category, representing patients in the initial steps of the metastatic cascade when the serum samples were collected, presented higher levels of several metabolites than the categories of patients who never developed metastases or had overt metastatic disease within 6 months of the primary tumour diagnosis (Fig. 2). The significantly elevated metabolites (Kruskal-Wallis test) were alanine (*P* = 0.005), lactate (*P* = 0.023), pyruvate (*P* = 0.041), citrate (*P* = 0.007) and creatinine (*P* = 0.047).

Serum citrate was elevated in the metachronous metastases group of patients who had presented with stage III disease as compared to the stage III subjects who did not have such outcome (*P* = 0.029, Mann-Whitney U test). Similarly, patients with stage I-II disease that progressed to metastasis had higher serum levels (Mann-Whitney U test) of alanine (*P* = 0.009), lactate (*P* = 0.046) and pyruvate (*P* = 0.024), as was also the case for citrate (*P* = 0.066), than the stage I-II subjects who did not. These observations show the added value of the identified metabolites beyond the traditional disease stage as metastatic risk factors.

**Fig. 3:**
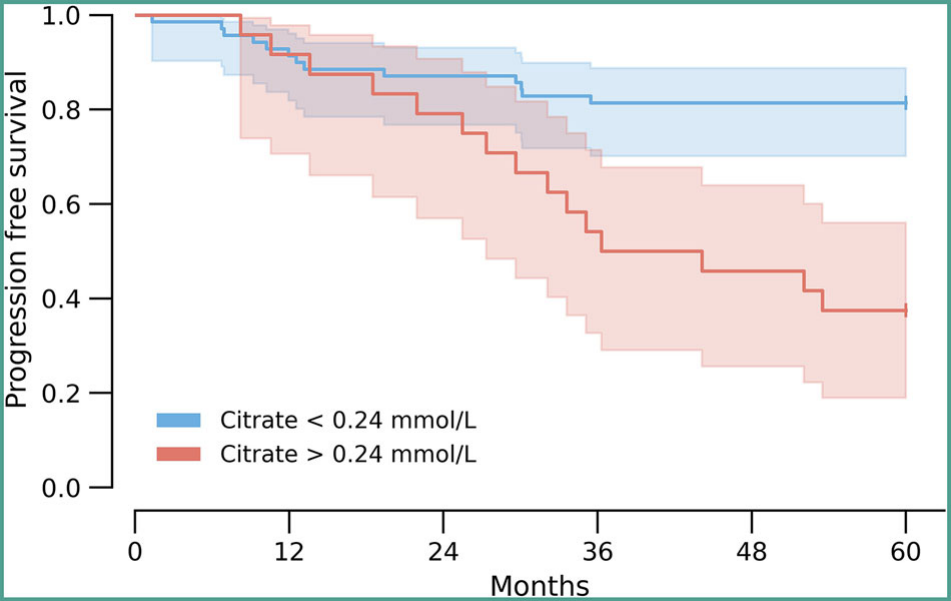
Progression-free survival for patients with serum citrate below or above the given cut-off value. Shading, 95% confidence interval; *P* < 0.001 (log-rank test).

Next, we assessed the prognostic impact of serum citrate in the groups of patients without overt metastases at the time of rectal cancer diagnosis (stage I-III; 94 of the 123 cases), using PFS as the end point and the best cut-off citrate value (0.24 mmol/L; upper reference limit 0.25 mmol/L for serum). Patients with citrate levels above the cut-off (*n* = 24) reached a median PFS 40.9 months (95% confidence interval, 33.7-48.2), while 81.4% of patients with citrate levels below (*n* = 70) had not experienced a progression event at this follow-up time. PFS for the latter patient group was clearly superior (*P* < 0.001, log-rank test; Fig. 3).

Patients with metastatic disease at the time of rectal cancer diagnosis (stage IV; 27 of the 123 cases) had significantly lower levels (Kruskal-Wallis test) of alanine (*P* = 0.041) and creatinine (*P* = 0.027), but no other of the assessed serum metabolites, compared to the patients presenting without metastases (Supplementary Fig. 2). Metastatic disease is commonly associated with loss of appetite, eventually resulting in debilitating muscle wasting^8^, which probably explains the low creatinine.

### Serum metabolic profiles and other patient and disease characteristics

A subgroup of 32 (of the 123) patients received neoadjuvant radiation (short-course radiotherapy or long-course chemoradiotherapy before surgical resection of the primary tumour, pursuant to national guidelines), with the surgical specimen scored for response in terms of tumour regression grade (TRG) ranging from the absence or sporadic presence of residual tumour cells (TRG 1) to minimal evidence of treatment response (TRG 3). TRG is a surrogate marker for metastasis risk^9^. Of note, the patients responding poorly to the neoadjuvant radiation (TRG 3 cases; *n* = 9), thus at elevated risk of progressing to metastatic disease, had significantly higher serum alanine levels at the time of diagnosis than the TRG 1 counterparts (*n* = 11; *P* = 0.048, Mann-Whitney U test; data not shown).

When analysing the serum ^1^H-NMR data for sex differences (Supplementary Fig. 3), several metabolites were higher (Mann-Whitney U test) in males (*n* = 79)—leucine (*P* = 0.002), isoleucine (*P* < 0.001), valine (*P* = 0.005), lysine (*P* = 0.005), glutamate (*P* < 0.001), pyruvate (*P* = 0.031), creatinine (*P* < 0.001) and phenylalanine (*P* = 0.045)—while creatine was higher in females (*n* = 44; *P* = 0.007, Mann-Whitney U test).

Systemic inflammation is a dominant attribute of advanced colorectal cancer (CRC), conferring poor outcome^10^, but the serum level of C-reactive protein (*n* = 117) was not correlated (Spearman’s rank correlation) with alanine, lactate, pyruvate or citrate. Similarly, no correlation (Spearman’s rank correlation) was found between serum carcinoembryonic antigen (*n* = 119), a common CRC biomarker used in routine practice, and any of the four metabolites.

Most of the serum metabolites were unaffected by patients’ concomitant medications, reflecting comorbidities, with the exception of the use of statins significantly elevating isoleucine, creatinine and α-glucose and of corticosteroids expectedly elevating α-glucose (Supplementary Table 1). As an internal methodological control, the 11 patients on metformin (a medication prescribed for type 2 diabetes) had significantly higher serum levels of α-glucose, but no other of the assessed metabolites, compared to the remaining rectal cancer patients (*P* = 0.011, Mann-Whitney U test; data not shown). Serum as biological matrix may in principle reflect any pathological alteration in the organism.

## Discussion

**Fig. 4:**
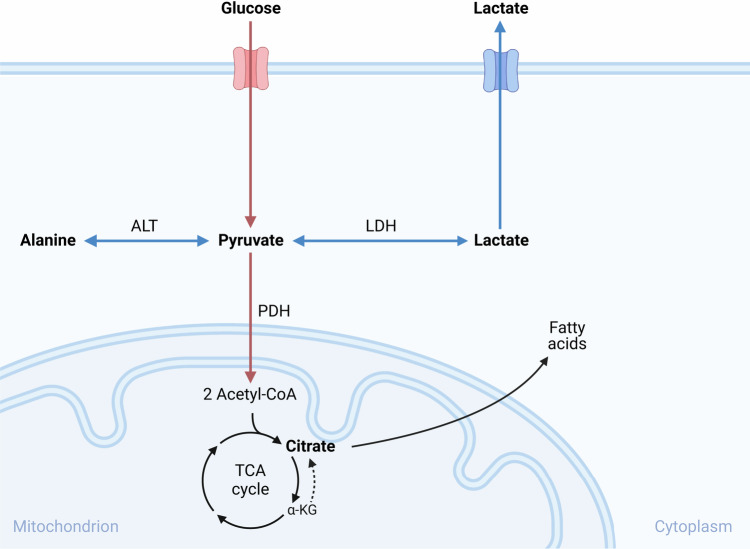
Simplified figure of the interconnection between pyruvate, lactate, alanine and citrate. α-KG α-ketoglutarate, ALT alanine aminotransferase, LDH lactate dehydrogenase, PDH pyruvate dehydrogenase, TCA tricarboxylic acid. The figure is created in BioRender.

The elevated serum metabolites might reflect the enhanced metabolic activity of primary tumour cells preparing to undertake the demanding activity of metastatic progression. Cancer cells display a distinctive metabolic phenotype characterised by increased glucose uptake and utilisation, even under normoxic tissue conditions, a phenomenon known as the Warburg effect. The significantly elevated metabolites alanine, lactate, pyruvate and citrate are metabolites of glycolysis (Fig. 4), where the glucose is catabolised to pyruvate that further may be converted to lactate by the enzyme lactate dehydrogenase or alanine by the enzyme alanine aminotransferase (ALT), both reversible reactions.

In normal cells most of the pyruvate enters the mitochondria as fuel for the tricarboxylic acid (TCA) cycle (Fig. 4), but cancer cells commonly hold increased lactate dehydrogenase activity, leading to high lactate and an acidic tumour microenvironment (TME)^11^. Lactate, previously viewed as a glycolysis by-product, promotes tumour aggressiveness and metastatic progression^12,13^. High serum pyruvate and lactate have been reported in CRC^14^ and suggested to support cancer cells’ successful transition through the blood circulation^3^.

Hypoxic tissue conditions, typical for solid tumours, lower the activity of the enzyme pyruvate dehydrogenase and thus the irreversible conversion of pyruvate for the mitochondrial TCA cycle, in favour of the competitor enzyme ALT and the conversion to alanine^15^. Of note, ALT may also metabolise pyruvate to α-ketoglutarate, a TCA cycle metabolite (Fig. 4) considered crucial for remodelling of the extracellular matrix in the metastatic niche when breast cancer cells colonise the lungs^16^. Alanine has also been shown to outcompete glucose as the primary fuel for the TCA cycle in hypoxic pancreatic ductal adenocarcinoma^17^, a highly aggressive gastrointestinal tract malignancy.

Citrate was the relatively most elevated serum metabolite in the metachronous metastases category, as compared to the patient categories without metastases at the 5-year follow-up or with metastases already 6 months or sooner after the diagnosis of rectal cancer. To facilitate tumour development, cancer cells not only have an increased demand for energy but also for the synthesis of macromolecules, in particular fatty acids for membrane biogenesis with citrate as the primary substrate^18^. Experimental model studies have shown that tumour cells import extracellular citrate, released from stromal TME cells, to support proliferation and metastatic progression^19^. The concept known as the Reverse Warburg effect suggests a reciprocal feeding of oxidative phosphorylation in the tumour cells by lactate from TME cells^20^. It has been hypothesised that citrate is the pivotal switch between glycolysis (the classic Warburg effect) and the oxidative phosphorylation^18^. Moreover, the TCA cycle may reverse itself by transforming α-ketoglutarate into citrate (Fig. 4) to meet the biosynthetic demands of fatty acids in hypoxic cancer cells^21^, particulary during metastasis^22,23^. This ability of cancer cells allows the maintenance of TCA cycle intermediates that support lipid biosynthesis and also provide energy. Citrate has thus been investigated in a number of preclinical settings, highlighting that the metabolic transition to extracellular supply under hypoxic and low glucose conditions enhances tumour progression. In this context and to the best of our knowledge, our study is the first indication of the role of citrate in patients who are essentially in the initial steps of the metastatic process.

Although some previous studies have analysed serum ^1^H-NMR spectra from CRC patients, we have not been able to identify other rectal cancer studies focusing on metabolomics in the metastatic process within a controlled, prospective setting. Previous studies have shown an increase in metabolites associated with glycolysis when comparing CRC patients with control subjects^14,24^.

A caveat in this study is the evident metabolic disparities between the sexes. Indeed, females and males showed significantly different levels of several metabolites. It was not surprising that creatinine, which is related to muscle mass, was higher in males, while creatine was higher in females as previously reported^25^. Although pyruvate was shown to be higher in males, there were no significant sex differences for alanine, lactate or citrate. Another remark is that patients were not necessarily fasting at blood sampling, adding another variable to the metabolic profile, but the results may thus be more representative for the routine clinical setting.

One study limitation was the resolvability of the ^1^H-NMR spectrum. Several metabolites can overlap in their chemical shift, further complicated by individual shift due to pH differences, and therefore be difficult to separate regardless of resolution of the obtained spectra. In particular, this may have influenced our measurements of glutamine, glutamate, valine, leucine and isoleucine. However, the main metabolites found in this study—alanine, lactate, citrate and to some extent pyruvate—were easily identifiable and resolvable for integration and quantification. Additional limitations were the restricted patient number of this study, particularly in the metachronous metastases group which reflects the improved rectal cancer outcome from contemporary therapies and the potential for further refinement, as well as possible confounding bias from using unadjusted statistical tests.

The absolute quantification of metabolites entails various uncertainties regarding the relaxation behaviour of the metabolites in relation to the standard TSP. We assumed for the absolute quantification that the relaxation behaviour was similar. However, the results presented do not rely on this absolute quantification and are valid even for relative measurements of the measured metabolites. Still, an analytical impact of differences in the relaxation behaviour among the patients categories (no, metachronous or synchronous metastases) can not be ignored. Further limitations of the ^1^H-NMR methodology were the use of D_2_O as solvent, a recent perspective has called attention to deuterium as a potential affector on the stability and structure of biomolecules^26^.

## Conclusion

This study showed a distinct serum metabolic profile in rectal cancer patients prone to metastatic progression already at the time when the primary tumour was diagnosed, offering insights into the metabolism of metastatic progression. The patients who developed overt metastatic disease more than 6 months after the primary tumour diagnosis (the metachronous metastases category) showed significantly elevated serum levels of key metabolites, in particular citrate, a finding supported by previous experimental model studies and now to the best of our knowledge demonstrated for the first time in patients. Patients with high serum citrate levels had poor PFS. The ability of ^1^H-NMR to provide detailed metabolic information from routine blood samples may be developed into non-invasive predictive and prognostic tools for cancer patients.

## Supplementary information

Supplementary Information

Description of Additional Supplementary Materials

Supplementary Data

Reporting summary

## Data Availability

^1^H-NMR spectra are available at figshare (https://figshare.com/) with accession number 19786. All other data is available upon reasonable request from the corresponding author. Source data for Fig. 2 and Supplementary Figs. 2 and 3 are given as a Supplementary Data.
