# Supplementary material for: Serum metabolite levels identify incipient metastatic progression of rectal cancer
Source: Commun Med (Lond). 2025 Apr 27;5:142. doi: 10.1038/s43856-025-00868-w (PMC12034819; doi:10.1038/s43856-025-00868-w)
Supplement: Supplementary file 1 — Supplementary Information [file 43856_2025_868_MOESM1_ESM.docx]

**Supplementary Information**

**Serum metabolite levels identify incipient metastatic progression of rectal cancer**

Kine M. Bakke^1,2,*,†^, Paula A. Bousquet^1,*^, Sebastian Meltzer^1^, Tonje Bjørnetrø^1^, Frode Rise^3^, Alistair L. Wilkins^4^, Kathrine Røe Redalen^5^ & Anne Hansen Ree^1,6^

^1^Department of Oncology, Akershus University Hospital, Lørenskog, Norway. ^2^Department of Physics and Computational Radiology, Oslo University Hospital, Oslo, Norway. ^3^Department of Chemistry, University of Oslo, Oslo, Norway. ^4^School of Science and Engineering, University of Waikato, Hamilton, New Zealand. ^5^Department of Physics, Norwegian University of Science and Technology, Trondheim, Norway. ^6^Institute of Clinical Medicine, University of Oslo, Oslo, Norway, ^*^These authors contributed equally: Kine M. Bakke, Paula A. Bousquet. ^†^corresponding author: k.m.bakke@medisin.uio.no


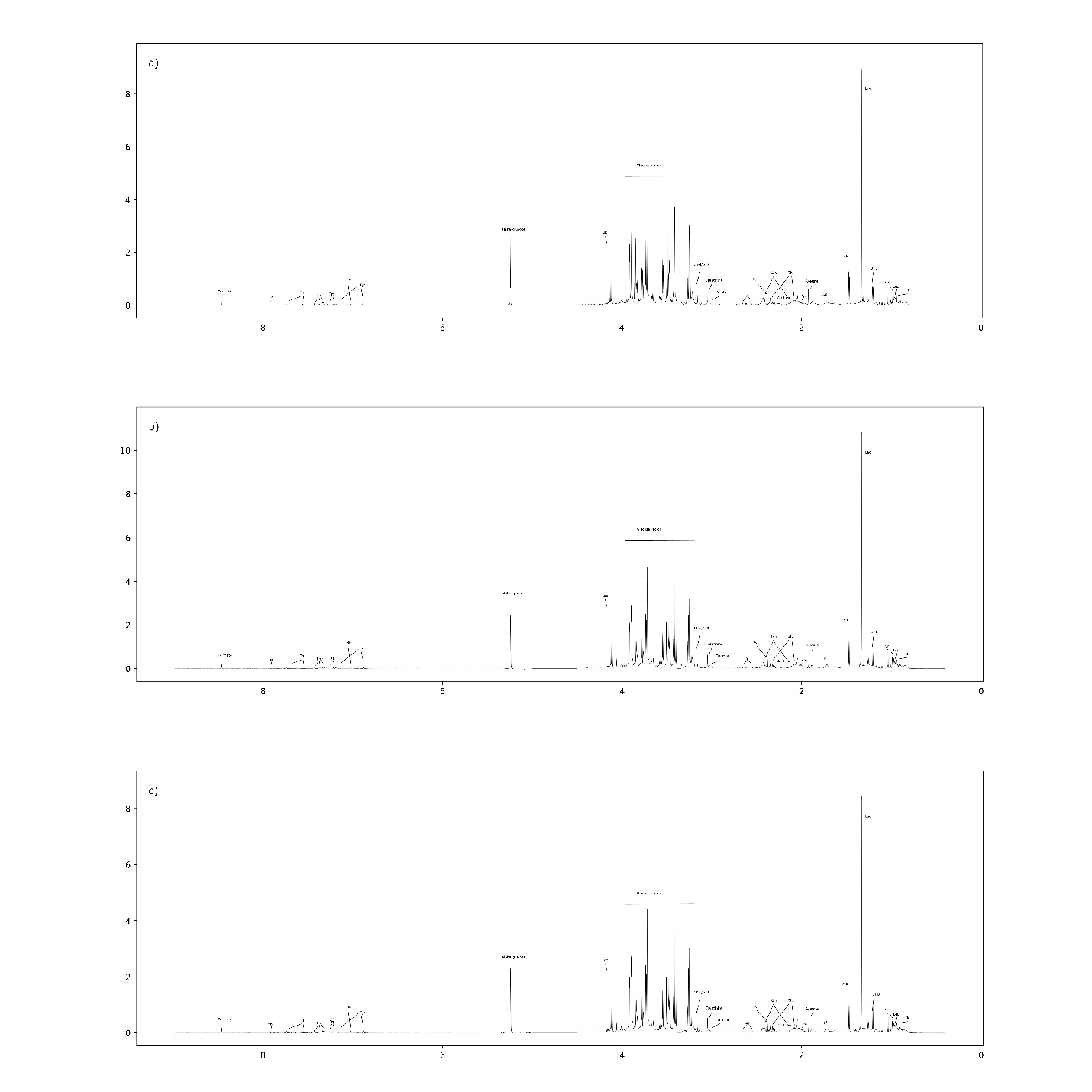


**Supplementary Figure 1. A comparison of NMR spectra for the groups with a) no metastases, b) metachronous metastases and c) synchronous metastases.** Abbreviations: 3HB, 3-hydroxybutyrate; Ala, alanine; Chol, choline; Cit, citrate; GPC, glycerophosphocholine; Gln, glutamine; Glu, glutamate; His, histidine; Ile, isoleucine; Lac, lactate; Leu, leucine; Lys, lysine; Phe, phenylalanine; Pro, proline; Pyr, pyruvate; Trp, tryptophan; Tyr, tyrosine; Val, valine.


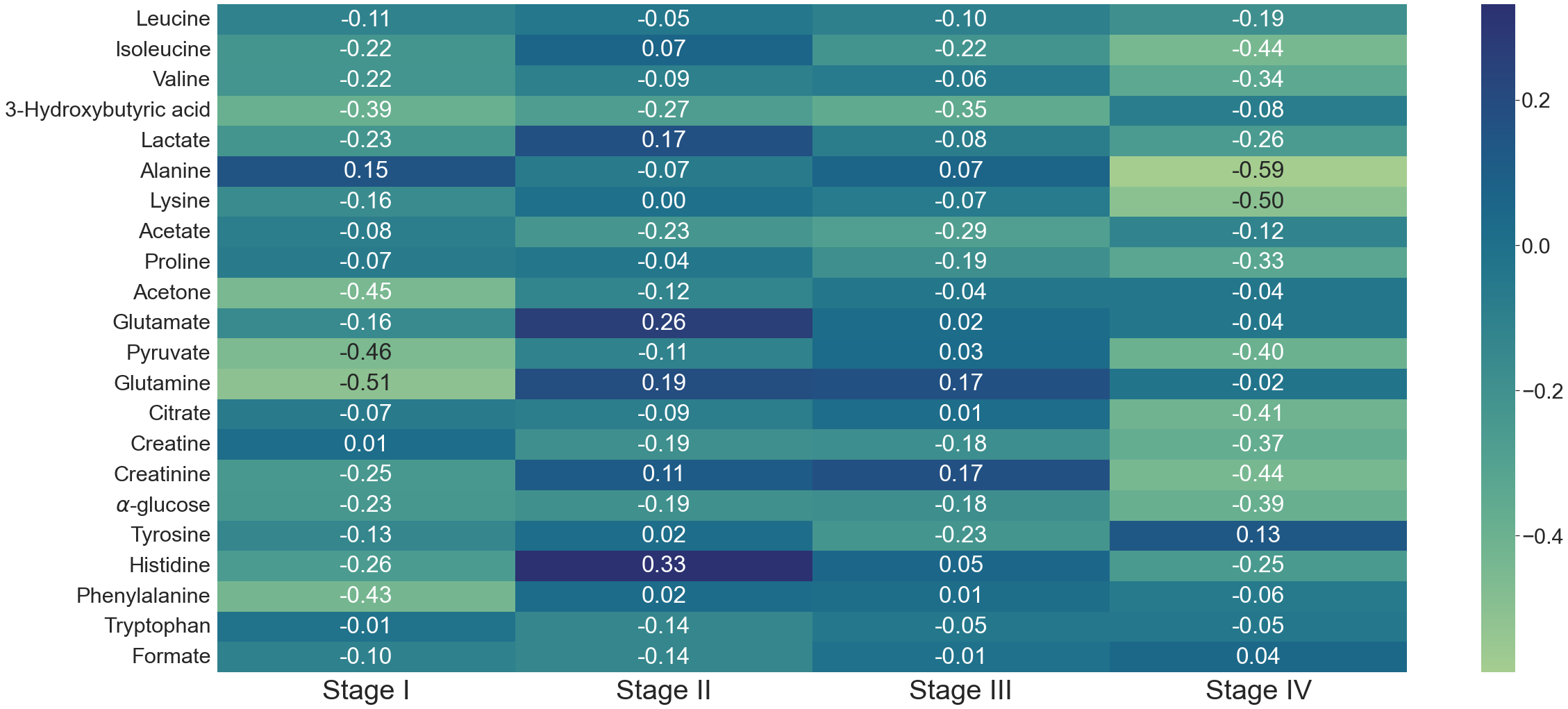
**Supplementary Figure 2. Serum metabolites according to rectal cancer stages.** The relative median metabolite values are shown for patients with stage I (*n* = 29), II (*n* = 29), III (*n* = 38) and IV (*n* = 27) disease. The individual metabolites have been z-normalised for the colour variation to illustrate the differences between the patient groups.


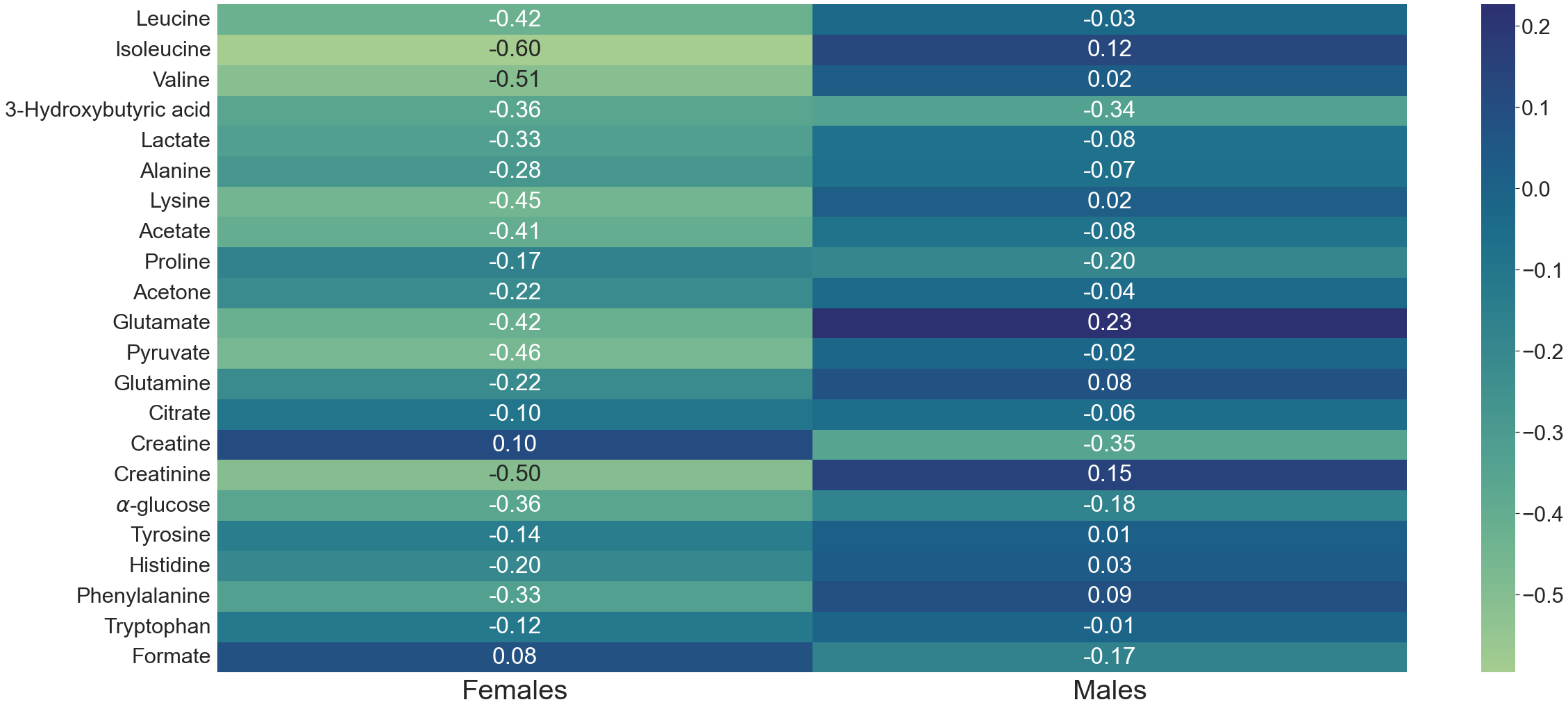


**Supplementary Figure 3. Serum metabolites according to sex.** The relative median metabolite values are shown for females (*n* = 44) and males (*n* = 79). The individual metabolites have been z-normalised for the colour variation to illustrate the differences between the patient groups.

| **Metabolite** | **Anti-coagulants** | **Morphine derivatives** | **Statins** | **Antacids** | **Corticosteroids** | **Anxiolytics and antidepressants** |
| --- | --- | --- | --- | --- | --- | --- |
| **Non-users,**  ***n* (%)** | **87**  **(70.7)** | **93**  **(75.6)** | **98**  **(79.7)** | **102**  **(82.9)** | **103**  **(83.7)** | **99**  **(80.5)** |
| Leucine | –0.05 | –0.21 | –1.89 | –0.90 | –0.14 | –0.59 |
| Isoleucine | –0.96 | –0.13 | **–2.40** | –0.55 | –0.74 | –1.00 |
| Valine | –0.24 | –0.14 | –1.76 | –0.99 | –0.58 | –1.02 |
| 3-Hydroxybulyric acid | –1.34 | –1.70 | –0.42 | –0.52 | –1.00 | –0.45 |
| Lactate | –1.02 | –1.37 | –0.37 | –0.63 | –0.97 | –0.80 |
| Alanine | –0.24 | –0.20 | –1.82 | –1.45 | –1.72 | –0.07 |
| Lysine | –0.34 | –0.40 | –1.75 | –0.89 | –0.03 | –0.91 |
| Acetate | –1.30 | –1.13 | –1.63 | –1.19 | –1.39 | –1.12 |
| Proline | –0.07 | –0.33 | –1.78 | –1.71 | –0.71 | –0.25 |
| Acetone | –0.75 | –0.19 | –0.38 | –0.65 | –0.84 | –0.27 |
| Glutamate | –0.81 | –0.67 | –1.48 | –0.48 | –0.36 | –0.05 |
| Pyruvate | –0.73 | –0.38 | –0.99 | –0.73 | –0.89 | –0.05 |
| Glutamine | –0.59 | –0.14 | –1.82 | –0.48 | –1.19 | –0.01 |
| Citrate | –0.12 | –0.68 | –1.40 | –0.85 | –0.15 | –0.13 |
| Creatine | –0.31 | –0.52 | –1.23 | –0.44 | –0.25 | –0.14 |
| Creatinine | –0.11 | –0.18 | **–2.06** | –0.42 | –1.10 | –1.40 |
| ⍺-glucose | –0.97 | –0.24 | **–2.83** | –0.15 | **–1.98** | –0.22 |
| Tyrosine | –1.91 | –1.02 | –1.30 | –1.06 | –1.32 | –0.11 |
| Histidine | –1.11 | –0.50 | –1.16 | –0.48 | –0.86 | –1.42 |
| Phenylalanine | –0.85 | –0.66 | –1.23 | –0.71 | –0.74 | –0.55 |
| Tryptophan | –1.24 | –0.55 | –0.72 | –0.75 | –0.30 | –0.14 |
| Formate | –0.22 | –0.86 | –0.08 | –0.82 | –0.007 | –1.14 |

**Supplementary Table 1. Serum metabolites according to use of concomitant medications**

The relative difference between the median group value for each metabolite in patients without the medications (non-users) *versus* those using the medications at the time of study entry. The six most frequent medication groups were chosen for comparison using Mann-Whitney U test. Significant differences are highlighted in bold.
