# Supplementary material for: Serum metabolite levels identify incipient metastatic progression of rectal cancer
Source: Commun Med (Lond). 2025 Apr 27;5:142. doi: 10.1038/s43856-025-00868-w (PMC12034819; doi:10.1038/s43856-025-00868-w)
Supplement: Supplementary file 2 — Description of Additional Supplementary Materials [file 43856_2025_868_MOESM2_ESM.pdf]

Description of Additional Supplementary Files

File name: Supplementary Data

Description: Source data for Figure 2 and Supplementary Figure 2 and 3
