# Supplementary material for: Serum metabolite levels identify incipient metastatic progression of rectal cancer
Source: Commun Med (Lond). 2025 Apr 27;5:142. doi: 10.1038/s43856-025-00868-w (PMC12034819; doi:10.1038/s43856-025-00868-w)
Supplement: Supplementary file 4 — Reporting summary [file 43856_2025_868_MOESM4_ESM.pdf]

nature portfolio

Corresponding author(s): K.M. Bakke, k.m.bakke@medisin.uio.no

Last updated by author(s): Feb 21, 2025

Reporting Summary

Nature Portfolio wishes to improve the reproducibility of the work that we publish. This form provides structure for consistency and transparency in reporting. For further information on Nature Portfolio policies, see our Editorial Policies and the Editorial Policy Checklist.

Statistics

For all statistical analyses, confirm that the following items are present in the figure legend, table legend, main text, or Methods section.

n/a	Confirmed	
☐	☒	The exact sample size (*n*) for each experimental group/condition, given as a discrete number and unit of measurement
☒	☐	A statement on whether measurements were taken from distinct samples or whether the same sample was measured repeatedly
☐	☒	The statistical test(s) used AND whether they are one- or two-sided<br>*Only common tests should be described solely by name; describe more complex techniques in the Methods section.*
☐	☒	A description of all covariates tested
☐	☒	A description of any assumptions or corrections, such as tests of normality and adjustment for multiple comparisons
☐	☒	A full description of the statistical parameters including central tendency (e.g. means) or other basic estimates (e.g. regression coefficient) AND variation (e.g. standard deviation) or associated estimates of uncertainty (e.g. confidence intervals)
☐	☒	For null hypothesis testing, the test statistic (e.g. *F*, *t*, *r*) with confidence intervals, effect sizes, degrees of freedom and *P* value noted<br>*Give P values as exact values whenever suitable.*
☒	☐	For Bayesian analysis, information on the choice of priors and Markov chain Monte Carlo settings
☒	☐	For hierarchical and complex designs, identification of the appropriate level for tests and full reporting of outcomes
☒	☐	Estimates of effect sizes (e.g. Cohen's *d*, Pearson's *r*), indicating how they were calculated

Our web collection on statistics for biologists contains articles on many of the points above.

Software and code

Policy information about availability of computer code

Data collection	1H-NMR spectra were obtained using a Bruker AVIIIHD800 spectrometer and the Topspin's (version 3.6; Bruker, Germany)
Data analysis	We have in the methods section referenced the use of the Matlab code Icoshift: Savorani F, Tomasi G, Engelsen SB. icoshift: A versatile tool for the rapid alignment of 1D NMR spectra. Journal of Magnetic Resonance. 2010;202(2):190-202

For manuscripts utilizing custom algorithms or software that are central to the research but not yet described in published literature, software must be made available to editors and reviewers. We strongly encourage code deposition in a community repository (e.g. GitHub). See the Nature Portfolio guidelines for submitting code & software for further information.

Data

Policy information about availability of data

All manuscripts must include a data availability statement. This statement should provide the following information, where applicable:

- Accession codes, unique identifiers, or web links for publicly available datasets
- A description of any restrictions on data availability
- For clinical datasets or third party data, please ensure that the statement adheres to our policy

1H-NMR spectra have been deposited at figshare (https://figshare.com/) with accession number 197863. All other data is available upon reasonable request from the corresponding author.

Human research participants

Policy information about studies involving human research participants and Sex and Gender in Research.

Reporting on sex and gender	We have used the term sex as appropriate in biological research and have reported on sex differences in the study.
Population characteristics	Patients had confirmed rectal adenocarcinoma of all stages. Of 123 patients, 44 were female, the average age was 65.
Recruitment	Unselected, all-commers
Ethics oversight	The study was approved by the Regional Committee for Medical and Health Research Ethics of South-East Norway.

Note that full information on the approval of the study protocol must also be provided in the manuscript.

Field-specific reporting

Please select the one below that is the best fit for your research. If you are not sure, read the appropriate sections before making your selection.

☒ Life sciences ☐ Behavioural & social sciences ☐ Ecological, evolutionary & environmental sciences

For a reference copy of the document with all sections, see nature.com/documents/nr-reporting-summary-flat.pdf

Life sciences study design

All studies must disclose on these points even when the disclosure is negative.

Sample size	Establishing a method for circulating biomarkers of rectal cancer aggressiveness was an explorative objective of the prospective observational biomarker study; hence, sample size was not calculated for the analysis of serum metabolites. The study was primarily designed for establishing a method for MR imaging biomarkers of rectal cancer aggressiveness.
Data exclusions	In three patients the obtained 1H-NMR spectra were technically defective and were excluded.
Replication	We collected and analysed serum samples from 123 unselected patients in a prospective observational biomarker study from a population-based patient cohort. We have not been successful in identifying a similar patient cohort that might be used to verify the reproducibility of the experimental findings.
Randomization	There was no randomization in the study
Blinding	The authors who performed the analyses were blinded to the metastatic status during the actual measurements, because this status was not apparent before the end of patient follow-up

Reporting for specific materials, systems and methods

We require information from authors about some types of materials, experimental systems and methods used in many studies. Here, indicate whether each material, system or method listed is relevant to your study. If you are not sure if a list item applies to your research, read the appropriate section before selecting a response.

Materials & experimental systems

n/a	Involved in the study	
☒	☐	Antibodies
☒	☐	Eukaryotic cell lines
☒	☐	Palaeontology and archaeology
☒	☐	Animals and other organisms
☐	☒	Clinical data
☒	☐	Dual use research of concern

Methods

n/a	Involved in the study	
☒	☐	ChIP-seq
☒	☐	Flow cytometry
☒	☐	MRI-based neuroimaging

Clinical data

Policy information about clinical studies

All manuscripts should comply with the ICMJE guidelines for publication of clinical research and a completed CONSORT checklist must be included with all submissions.

Clinical trial registration	NCT01816607
Study protocol	https://clinicaltrials.gov/study/NCT01816607
Data collection	Patients were unselected all-comers within the public specialist health service in Norway. Patients were recruited onto the biomarker study between October 2013 and December 2017 and were followed for 5 years after the primary treatment of rectal cancer for clinical data collection.
Outcomes	The pre-defined primary outcome was progression-free survival, which was measured by periodic CT scans according to clinical routine practice and national guidelines. No secondary clinical outcomes are reported.
